# A Call to Change the Nomenclature of “Open” Inguinal Hernia Repair

**DOI:** 10.3389/jaws.2024.13868

**Published:** 2025-01-03

**Authors:** Kaela Blake, Nora Fullington, Michael Reinhorn

**Affiliations:** ^1^ University of Tennessee Graduate School of Medicine, Knoxville, TN, United States; ^2^ Boston Hernia, Wellesley, MA, United States; ^3^ Mass General Brigham-Newton Wellesley Hospital, Newton, MA, United States; ^4^ School of Medicine, Tufts University, Boston, MA, United States

**Keywords:** inguinal hernia repair, laparoscopic inguinal hernia repair, MIS hernia repair, OPP, open preperitoneal inguinal hernia repair

In this edition of JAWS, many researchers have described numerous benefits of open preperitoneal (OPP) inguinal hernia repair. Overwhelming data suggests OPP inguinal hernia repair is a structurally sound, cost-effective approach for inguinal hernia repair with negligible rates of chronic groin pain and hernia recurrence [1–17]. The 2023 HerniaSurge Guidelines state that “open preperitoneal mesh techniques can achieve favorable results in terms of operating time, acute and chronic postoperative pain and return to work compared to Lichtenstein repair [17].” This is based on several recent randomized controlled trials that favor OPP to Lichtenstein for decreased pain and quicker recovery [18–21]. The guidelines also found that OPP and laparo-endoscopic approaches have comparable outcomes in terms of postoperative pain, recurrences and recovery, citing three randomized controlled trials [22–24]. Thus, OPP has outcomes that more similarly resemble those of Minimally Invasive Surgery (MIS) inguinal hernia repair [14, 17, 22–24] as opposed to Lichtenstein repairs.

Although OPP outcomes are more similar to those of MIS approaches, OPP is often categorized with Lichtenstein and tissue-based repairs in the broad category of “open” inguinal hernia repair [15]. We believe that categorizing these vastly different approaches together makes data collection and interpretation very difficult, leaving the surgical community unable to make clinically meaningful changes to improve patient outcomes. Furthermore, there are advantages of OPP compared to MIS approaches, such as decreasing cost, avoiding MIS equipment, and providing the opportunity to avoid general anesthesia [10, 14, 25–37]. We consider open preperitoneal repairs less invasive than the standard MIS operations as they do not enter the peritoneal cavity and are performed through one 3–4 cm incision instead of multiple incisions. The current standard, particularly in the United States, requires MIS equipment and general anesthesia to perform a preperitoneal inguinal hernia repair. In our view, this has created a platform for surgeons and device companies to market expensive technologies that may offer little to no benefit to individual patients while detrimentally increasing the cost of healthcare within our society. OPP provides a solution to this dilemma but needs more widespread acceptance, training opportunities and dedicated research with appropriate classification efforts to increase evidence-based recommendations.

The first step to distinguishing the benefits of OPP compared to other inguinal hernia repair techniques requires that the surgical community change the nomenclature regarding “open” inguinal hernia repairs. We have already done this for laparoscopic and robotic hernia surgery. We identify procedures by the anatomical planes, technology used, and location of mesh placement. We use terms like TAPP, TEP, and rTAPP to describe repairs that use laparoscopic or robotic technology to either enter the peritoneal cavity or stay in the pre-peritoneal plane. All of these procedures place mesh in the preperitoneal space and are commonly grouped together as “MIS” approaches in studies and publications. Similarly, several inguinal hernia repair techniques exist using an “open” approach. However, as previously mentioned, these approaches are significantly different from one another – both in planes dissected and placement of mesh - and have expectedly different outcomes. These open techniques must be clearly delineated in the literature and accepted in our surgical community in order to unify research efforts and guidelines. Therefore, we propose the following categorization of open inguinal hernia repair approaches:

• “Open tissue (OT)” repairs: This dissection occurs in the space below the external oblique aponeurosis and superficial to the pre-peritoneal space. These repairs include Bassini, Shouldice, Desarda and others.
• “Open Anterior Mesh (OAM)” repairs: This uniquely describes an anterior onlay mesh above the internal oblique musculature and deep to the external oblique aponeurosis, classically known as the Lichtenstein repair.
• “Open preperitoneal (OPP)” repairs: Describes open approaches where mesh is placed behind the abdominal wall, in the pre-peritoneal space. Examples include: TIPP, MOPP, TREPP, Kugel and various permutations of these repairs.
• “Open Anterior and Posterior Mesh (OAPM)” repairs: Although discouraged in international guidelines, many surgeons still utilize a hybrid technique where mesh is placed in both the anterior and posterior planes, such as Prolene Hernia System and Plug and Patch.

It is crucial that we correct the generalization that all “open” inguinal hernia repairs are equal. We must also overcome the marketing barrier that preperitoneal repairs require a laparoscope or robot. Only then can we objectively review the outcomes associated with various repairs, and identify specific operations that offer the best value to our patients, institutions and society as a whole.
